# Association of sleep disorders with clinical symptoms and age in Chinese older adult patients with and without cognitive decline

**DOI:** 10.3389/fnagi.2023.1189837

**Published:** 2023-08-09

**Authors:** Xi Mei, Zheng Zhao, Zhengfa Qiu, Juan Wang, Haihang Yu, Chengying Zheng

**Affiliations:** Department of Psychiatry, Ningbo Kangning Hospital and Affiliated Mental Health Centre, Ningbo Key Laboratory for Physical Diagnosis and Treatment of Mental and Psychological Disorders, Ningbo University, Ningbo, Zhejiang, China

**Keywords:** sleep disorders, cognitive decline, aging, dementia, oxygen saturation

## Abstract

**Objective:**

To investigate correlation between cognitive function, age, and sleep disturbances.

**Methods:**

This retrospective clinical study enrolled 78 patients with sleep disorders who were divided into three groups: a group of 24 patients with sleep disorders accompanied by cognitive decline (SD-CD); 54 patients with sleep disorders and no cognitive decline (SD-nCD) was divided into two groups, one of 30 patients aged between 60 and 70 years and another of 24 patients aged >70 years. Polysomnography was used to record patients’ sleep indicators throughout night; these included total sleep duration, sleep efficiency (SE), sleep latency, sleep structure and percentage of N1, N2, and N3 stages, rapid eye movement (REM) stage, as well as apnea hypopnea index (AHI), and oxygen saturation (OS). Analysis of variance (ANOVA) for continuous variables and chi-square test for categorical variables were used to analyze variables between different groups. Pearson’s correlation was used to analyze correlation between sleep parameters and mini-mental state examination (MMSE). Blood samples were used to determine their Aβ, Aβ_40_, Aβ_42_, total tau, phosphorylated tau protein (ptau), ptau_181_, ptau_217_, the inflammatory factor IL-1β, vitamin B12 (VB12), and melatonin levels.

**Results:**

In the SD-CD group, there was a significant decrease in SE and an increase in N1 stage sleep in older patients and a significant increase in AHI, REM stage AHI, and non-REM stage AHI. In patients with SD-nCD, the minimum OS, minimum OS in the REM period, and minimum OS in the non-REM period were significantly reduced. OS was significantly correlated with cognitive level, as evaluated by the MMSE. The addition of sleep parameters can significantly improve the accuracy of dementia diagnosis. Dementia biomarkers of Aβ and tau proteins in blood showed cognition-related differences, while ptau181 was associated with both cognition and age-related differences. Regression models revealed that age was related to higher levels of cognitive decline before (β = −0.43, *P* < 0.001) and after (β = −0.38, *P* < 0.001) adjustment of gender, BMI, and education level. There was a significant mediation effect of relationship between aging and cognitive function by sleep efficiency and N1 stage sleep.

**Conclusion:**

Sleep disorders and low OS are associated with a higher incidence of cognitive decline and dementia.

## 1. Introduction

Aging is associated with a decline in sleep quality and cognitive function. Sleep architecture changes with age; for example, there is a decrease in the slow wave sleep (SWS/N3) stage as well as a reduction in rapid eye movement (REM) periods (Mander et al., 2017). These changes are divided into normal aging, which generally does not affect cognitive function, and pathological changes, which can affect cognitive function to some extent. However, there is also a correlation between reduced sleep quality and cognitive decline. This correlation is commonly observed in older adults with normal sleep, insomnia, or sleep breathing disorders, as well as in those with normal cognition or dementia (Dzierzewski et al., 2018). The age factor should also be considered because of the prevalence of both sleep-disordered breathing and cognitive changes that increase with age (Ayalon et al., 2010; Peppard et al., 2013).

Aside from aging, previous studies have found that a proportion of patients with sleep disorders experience cognitive decline (Cricco et al., 2001). Older adults with long-term insomnia and long-term use of hypnotics had a two-fold higher risk of developing dementia during a 3-year follow-up period than healthy controls (Chen et al., 2012). There may be a physiological mechanism by which lack of sleep increases the accumulation of Aβ in the brain; conversely, proper sleep reduces the generation of Aβ and enhances its clearance (Xie et al., 2013). Sleep disruption and circadian rhythm disorders often occur in patients with cognitive impairments. Circadian dysfunction and sleep disturbances are the most common features in patients with Alzheimer’s disease (AD) (Musiek et al., 2015). The existence of disturbed sleep during preclinical and clinical disease development emphasizes the central role of sleep in the pathogenesis and development of dementia (Uddin et al., 2020).

Furthermore, the prevalence of sleep disorders in patients with dementia is high, and the common ones are REM sleep behavior disorder, increased daytime sleepiness, and sleep breathing syndrome (Wennberg et al., 2017). Approximately 45% of patients with AD have sleep difficulties. These symptoms may persist for several years prior to the medical diagnosis of AD (Urrestarazu and Iriarte, 2016). According to the above evidence, it has strong relationship between age, sleep disorders, and cognitive decline. But the association of sleep disorders with clinical symptoms and age in Chinese older adult patients with and without cognitive decline was unclear.

In this study, we aimed to investigate the correlation between age, sleep quality and cognitive function. Three groups of patients were selected. One consisted of patients with sleep disorders accompanied by cognitive decline (SD-CD) and the other of patients with sleep disorders alone (sleep disorder with no cognitive decline, SD-nCD). The latter were further divided into two groups of old and young according to age. In the SD-CD group and SD-nCD old group, there was no significant difference in the mean age of the subjects. It was used to find indicators of differences other than the age factor. We also compared the SD-nCD old and younger groups to see the effect of the age factor. The clinical characteristics, sleep parameters, and blood indicators of the three groups were analyzed. The differences between the groups, especially those parameters that correlated more with cognitive decline, could be used as biomarkers to assess future cognitive decline. The potential benefits of sleep treatments on cognitive function can also be used as sleep intervention targets for the prevention of cognitive decline.

## 2. Materials and methods

### 2.1. Clinical participants

The 78 patients with sleep disorders with or without cognitive decline were diagnosed using the Diagnostic and Statistical Manual of Mental Disorders, fifth edition (DSM-V) criteria (Battle, 2013). All of the patients had to meet the following inclusion criteria: (1) they met the sleep disorder diagnosis made by two research psychiatrists according to the DSM-V; (2) they were able to provide informed consent; (3) their disease course was >3 months; (4) if they had severe cognitive decline they used the same cholinesterase inhibitor, donepezil. The exclusion criteria for patient participants were (1) they had other severe mental illnesses or history, including schizophrenia, bipolar disorder, delirium and (2) severe physical diseases such as cancer. Anxiety and depression were not in the exclusion criteria. The group of SD-CD consisted of age- and gender- matched patients reporting sleep disorders and was confirmed by Pittsburgh sleep quality index (PSQI) > 7. The neuropsychological evaluation of cognitive decline was confirmed by mini-mental state examination (MMSE) score <17, 20, and 24 in person with education levels of illiteracy, primary school, and junior high school, respectively.

### 2.2. Participants recruitment

**FIGURE 1:**
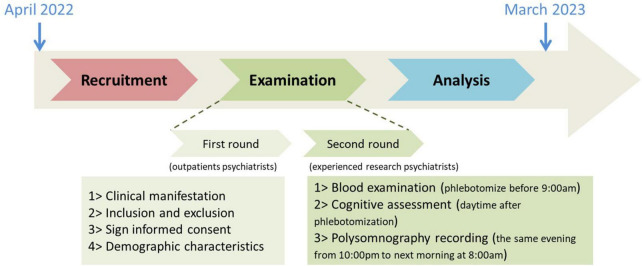
Schematic diagram of the study.

We recruited inpatients and outpatients at Sleep Medicine Center of Ningbo Kangning Hospital between April 2022 and March 2023. Two screening rounds were conducted as shown in Figure 1. In the first round, geriatric outpatient psychiatrists conducted a preliminary screening. In the second round, patients who may meet the inclusion criteria were recommended to experienced research psychiatrists. This study was approved by the ethics committee of Ningbo Kangning Hospital. All participants or their legal guardians signed informed consent forms. Demographic characteristics include age, gender, body mass index, and educational level.

### 2.3. Cognitive assessment

The mini-mental state examination (MMSE) consists of 11 cognitive questions in the areas of orientation, immediate recall, attention, short-term memory, and language. The MMSE has a maximum score of 30, with higher scores indicating better cognitive performance.

### 2.4. Polysomnography recording

According to standard procedures, polysomnography (PSG) of the participants was recorded from 22:30 pm to 06:30 am in a sleep-monitoring ward. The participants were required to arrive at least 2 h before sleep monitoring to become familiar with the environment. The PSG records were maintained in accordance with the guidelines of the American Academy of Sleep Medicine (Kushida et al., 2005). All recordings included electroencephalograph leads, bilateral electrooculogram leads, submentalis electromyogram leads, and electrocardiograms.

A professional technician evaluated the PSG data according to standard criteria (Malhotra et al., 2018). We collected data on sleep continuity and sleep architecture, including total sleep time (TST), percentage of sleep efficiency (SE), sleep onset latency (SOL), rapid eye movement (REM) latency, percentages of stage 1 sleep (N1), percentages of stage 2 sleep (N2), percentages of slow wave sleep (SWS/N3), and percentages of REM sleep. We also collected data on the apnea hypopnea index (AHI) and oxygen saturation (OS).

### 2.5. Blood collection and plasma processing

Blood samples were collected before breakfast by phlebotomists who had experience with older patients. Using a winged blood collection set, 5 ml of whole blood was collected in a procoagulant tube. To allow the measurement of plasma peptides, blood was immediately centrifuged (1400 rpm using a BY-600A type medical centrifuge, Beijing Baiyang Medical Devices Co., China) for 10 min. All blood samples were processed within 30 min of collection and immediately frozen at −80°C.

Blood samples were thawed immediately before analysis. Serum amyloid-β (Aβ), Aβ_40_, Aβ_42_, total tau, phosphorylated tau (ptau), ptau_181_, ptau_217_, IL-1β, vitamin B 12 (VB_12_), melatonin levels were estimated using ELISA kits (Shanghai Yuanye Bio-Technology Co., China). All procedures were performed according to the manufacturer’s instructions. Absorbance was measured at 450 nm using a Sunrise-basic enzyme labeling instrument (Tecan Co., Switzerland) with a reference wavelength of 690 nm. These measurements were transformed into concentrations by comparing the optical densities of the samples with the standard curve values.

### 2.6. Statistical analysis

For statistical analysis, data are presented as mean ± standard deviation (SDs). Comparisons of the demographic and clinical variables between the different groups were analyzed using analysis of variance (ANOVA) for continuous variables and a chi-squared test for categorical variables. Pearson’s correlation was used to analyze the correlation between sleep quality and the cognitive assessment scale. Statistical significance was set at *p* < 0.05. Statistical Package for the Social Sciences (SPSS version 19.0, IBM) was used for all analyses.

The mediation models were made separately for sleep efficiency and N1 stage sleep. This included the following steps: (a) Prediction of the dependent variable (cognitive decline) by the independent variable (aging). (b) Prediction of the mediators (sleep efficiency and N1 stage sleep) by the independent variable (aging). (c) Prediction of the dependent variable (cognitive decline) by both the independent variable (aging) and the mediators (sleep efficiency and N1 stage sleep; for partial mediation it is required that the direct relation between the independent variable and the dependent variable is reduced by inclusion of the mediator to the model) (Preacher and Hayes, 2008). To also formally test the significance of the indirect effects we then additionally employed the SPSS procedure Indirect by Preacher and Hayes (2008), assessing the models with both mediators simultaneously via bootstrapping. Number of bootstrap samples for bias corrected bootstrap confidence intervals was 10,000.

## 3. Results

### 3.1. Clinical assessment

The characteristics of the patients included in this study are summarized in Table 1. There were 24 patients with SD-CD, including 9 men and 15 women. The older group of patients with sleep disorder but without cognitive decline (SD-nCD) consisted of 24 patients (9 men and 15 women) with a mean age of 74.0 years, while the younger group was made up 30 patients (13 men and 17 women) with a mean age of 63.5 years. Eight patients with SD-CD were taking sleep-related medications and twenty-one were taking cognitive improvement medications. Fifteen in the older group and twenty in the younger group of patients with SD-nCD were taking sleep medications.

**TABLE 1:** Demographics and clinical characteristics of study participants.

Characteristics		SD-D group	SD-nD group		F/χ^2^	*P*
		Older	Younger			
Number		24	24	30	0.923	0.630
Sex (Men/Women)		9/15	9/15	13/17	3.282	0.070
Age (years)		73.5 ± 8.7	74.0 ± 3.7	63.5 ± 3.4	30.406	<0.001
BMI		21.5 ± 3.1	22.7 ± 2.5	22.3 ± 2.6	1.272	0.286
Education (years)		6.0 ± 2.1	6.1 ± 1.1	6.4 ± 1.2	0.583	0.560
Sleep disorder type	SSD	15	23	30	12.159	<0.001
	SSD accompanied by OSA	9	1	0	12.159	<0.001
Dementia duration		3.4 ± 1.85	0	0	NA	NA
Hypertension		18	17	12	4.550	0.014
Diabetes mellitus		6	7	4	1.073	0.347
Sleep medicine		19	23	30	4.743	0.011
Dementia medicine		21	0	0	178.273	<0.001
PSQI		13.7 ± 4.27	12.0 ± 5.59	11.67 ± 5.98	0.198	0.820
MMSE		13.0 ± 6.8	25.7 ± 4.2	26.9 ± 3.3	63.994	<0.001
Drug (sleep)		8	15	20	5.997	0.017
Drug (cognition)		21	1	1	91.323	<0.001

Means ± SDs. SD-D, patients with dementia and sleep disorder; SD-nD, patients with no dementia and sleep disorder; BMI, body mass index; MMSE, Mini-Mental State Examination; SSD, sleep structure disorders; OSA, obstructive sleep apnea.

### 3.2. PSG analysis

**FIGURE 2:**
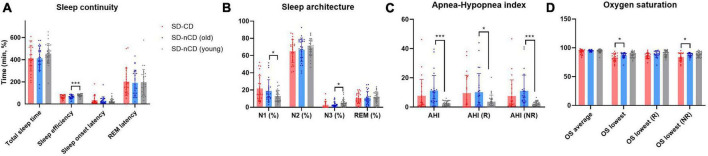
Polysomnography (PSG) parameters (**A**, sleep continuity; **B**, sleep architecture; **C**, apnea-hypopnea index; **D**, oxygen saturation) of three groups of patients with SD-CD (red), SD-nCD older group (blue), and SD-nCD younger group (gray). *p < 0.05, ***p < 0.001.

As shown in Figure 2 and Table 2, we found significant differences in SE (*F* = 7.191, *p* = 0.001), N1 (*F* = 3.757, *p* = 0.028), AHI (*F* = 7.014, *p* = 0.002), AHI (REM, R) (*F* = 3.473, *p* = 0.036), AHI (No REM, NR) (*F* = 6.968, *p* = 0.002), lowest OS (*F* = 5.049, *p* = 0.009), lowest OS (R) (*F* = 6.553, *p* = 0.002), and lowest OS (NR) (*F* = 5.858, *p* = 0.004) among the three groups. In terms of sleep continuity, the younger SD-nCD group had significantly higher SE than the other two groups. In terms of sleep architecture, the proportion of N1 stage sleep was significantly higher in the SD-CD group than in the other two groups. AHI was significantly higher in the older SD-nCD group than in the younger group. For minimum blood OS, blood oxygen was significantly lower in the SD-CD group than in the SD-nCD group.

**TABLE 2:** Polysomnographic parameters of study participants.

Characteristics	SD-D group	SD-nD group		F/?^2^	*P*
		Older	Younger		
**Sleep continuity**					
TST, min	414.1 ± 115.8	417.6 ± 92.8	425.0 ± 138.9	0.059	0.943
SE,%	66.7 ± 16.7	66.3 ± 15.0	79.4 ± 12.4	7.191	**0.001**
SOL, min	34.0 ± 40.1	26.4 ± 18.4	26.9 ± 21.7	0.750	0.476
REM latency, min	201.7 ± 123.8	193.2 ± 115.5	197.1 ± 114.9	0.031	0.969
**Sleep architecture, %total sleep time**					
N1, %	21.6 ± 15.0	19.1 ± 13.2	13.1 ± 6.7	3.757	**0.028**
N2, %	65.1 ± 13.2	67.1 ± 13.4	69.7 ± 9.8	0.998	0.374
SWS, %	2.7 ± 4.2	3.0 ± 3.1	5.0 ± 4.1	2.891	0.062
REM, %	10.5 ± 5.9	10.7 ± 7.3	12.2 ± 6.0	0.539	0.586
**Apnea-hypopnea index (AHI)**					
AHI	7.7 ± 11.2	11.2 ± 10.4	2.6 ± 1.7	7.014	**0.002**
AHI (R)	9.6 ± 12.1	10.3 ± 12.7	3.7 ± 4.4	3.473	**0.036**
AHI (NR)	7.5 ± 11.1	11.1 ± 10.8	2.3 ± 1.6	6.968	**0.002**
**Oxygen saturation (OS)**					
OS average	93.3 ± 4.1	94.8 ± 1.5	94.2 ± 2.9	1.402	0.253
OS lowest	83.3 ± 6.9	87.0 ± 4.8	88.1 ± 4.9	5.049	**0.009**
OS lowest (R)	73.9 ± 31.3	89.4 ± 5.7	90.3 ± 5.0	6.553	**0.002**
OS lowest (NR)	84.1 ± 7.3	87.6 ± 3.4	88.9 ± 4.2	5.858	**0.004**

Means ± SDs. D-SD, patients with dementia and sleep disorder; nD-SD, patients with no dementia and sleep disorder; TST, total sleep time; SE, sleep efficiency; SOL, sleep onset latency; REM, rapid eye movement; AHI, apnea-hypopnea index; OS, oxygen saturation. The bold values represent statistical significance (p < 0.05).

### 3.3. Subgroups analysis of younger SD-nCD patients

**FIGURE 3:**
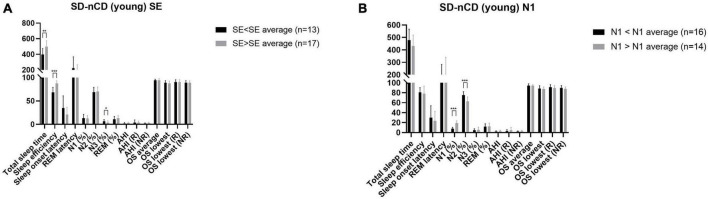
Polysomnography parameters (**A**, SE subgroup; **B**, N1 subgroup) of the younger group of patients with SD-nCD. Gray bar represent the values larger than average. Black bar represent the values less than average. *p < 0.05, **p < 0.01, ***p < 0.001.

The sleep parameters SE and N1 were significantly different between the younger and older SD-nCD groups. As shown in Figure 3, analysis of data from the younger SD-nCD patients revealed that when the group was divided into SE < SE average and SE > SE average groups, there were significant differences in the TST and SWS percentages between the two groups, in addition to significant differences in SE. When the group was divided into N1 < N1 average and N1 > N1 average groups, there were significant differences in the N2 percentages between the two groups. Statistical analysis was performed using the *t*-test.

### 3.4. Pearson correlation between cognitive and sleep parameters

**FIGURE 4:**
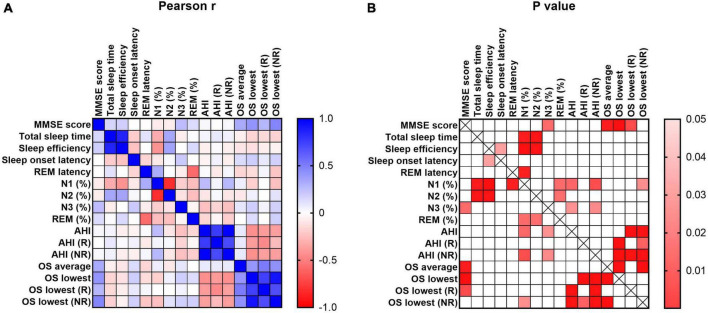
Pearson correlation of cognitive and sleep parameters. MMSE represents the cognitive level of participants. The sleep parameters included total sleep time, sleep efficiency, sleep onset latency, REM latency, percent of N1, N2, N3 and REM, AHI in REM stage and NREM stage, OS average and lowest value of REM and NREM stage. The scale bar represents the Pearson coefficient r and *P*-values.

As shown in Figure 4, MMSE scores were significantly correlated with SWS, average OS, lowest OS, and lowest OS (R) (*r* = 0.256, *p* = 0.024, *r* = 0.330, *p* = 0.004, *r* = 0.414, *p* < 0.001, *r* = 0.280, *p* = 0.016, respectively).

### 3.5. Receiver operating characteristic curve

**FIGURE 5:**
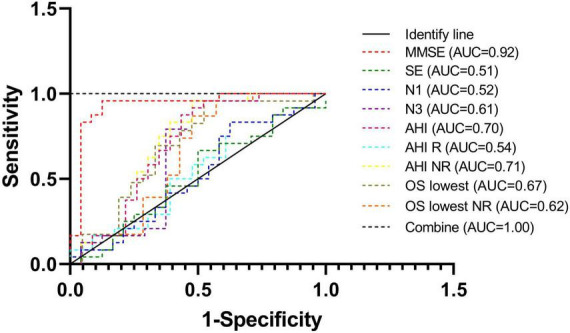
Receiver operating characteristic curves of different parameters of diagnosis. Combination of cognitive parameter and sleep parameters can improve the sensitivity and specificity.

For the SD-CD and older SD-nCD groups, the mean ages of the patients in both groups were matched. The sleep parameters of the two groups could be used to enhance the accuracy of dementia diagnosis. In Figure 5, as seen by the receiver operating characteristic (ROC) curve, the area under the curve (AUC) was significantly higher after combining multiple sleep indicators than with a single cognitive indicator.

### 3.6. Blood parameters

**FIGURE 6:**
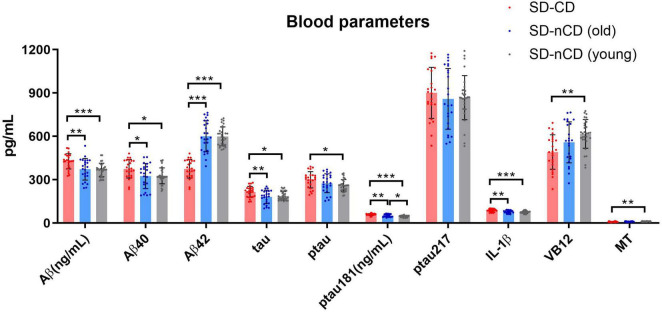
Blood parameters in the SD-CD and SD-nCD groups. The parameters include cognitive biomarkers of total Aβ, Aβ40, Aβ42, tau, Phosphorylated tau, Phosphorylated tau181 and 217, the inflammatory factor, Vitamin B12 and melatonin. *p < 0.05, **p < 0.01, ***p < 0.001.

We tested cognitive-related indicators in blood, including Aβ, Aβ_40_, Aβ_42_, total tau, phosphorylated tau protein (ptau), ptau_181_, ptau_217_, inflammatory factor IL-1β, vitamin B_12_ (VB_12_), and melatonin (Table 3). In the SD-CD group, aside from ptau_217_, the values of other parameters were significantly higher than those of the SD-nCD group. In the SD-nCD group, ptau_181_ expression was higher in the older subgroup than in the younger subgroup (Figure 6).

**TABLE 3:** Blood parameters of study participants.

Blood parameters	SD-D group	SD-nD group		*F*	*P*
		Older	Younger		
Aβ, ng/mL	426.99 ± 52.72	372.04 ± 74.88	367.47 ± 47.28	8.032	**0.001**
Aβ_40_, pg/mL	375.89 ± 64.75	324.62 ± 86.47	326.50 ± 55.14	4.414	**0.015**
Aβ_42_, pg/mL	695.20 ± 71.90	600.95 ± 107.33	599.55 ± 65.91	11.037	**0.000**
tau, pg/mL	215.75 ± 38.01	181.57 ± 45.98	187.90 ± 29.95	5.615	**0.005**
ptau, pg/mL	299.07 ± 55.97	275.25 ± 65.08	260.17 ± 41.32	3.470	**0.036**
ptau_181_, ng/mL	59.07 ± 6.67	51.64 ± 9.27	46.37 ± 5.96	20.032	**0.000**
ptau_217_, pg/mL	900.81 ± 176.20	859.30 ± 209.88	867.75 ± 152.39	0.367	0.694
IL-1β, pg/mL	87.69 ± 9.74	78.14 ± 9.97	76.01 ± 8.24	11.512	**0.000**
VB_12_, pg/mL	491.53 ± 118.55	558.41 ± 142.06	617.38 ± 99.93	7.358	**0.001**
melatonin, pg/mL	8.73 ± 2.50	9.34 ± 2.47	10.81 ± 2.02	5.831	**0.004**

Means ± SDs. D-SD, patients with dementia and sleep disorder; nD-SD, patients with no dementia and sleep disorder; Aβ, amyloid β; VB, vitamin B. The bold values represent statistical significance (p < 0.05).

### 3.7. Mediation effect analysis

**FIGURE 7:**
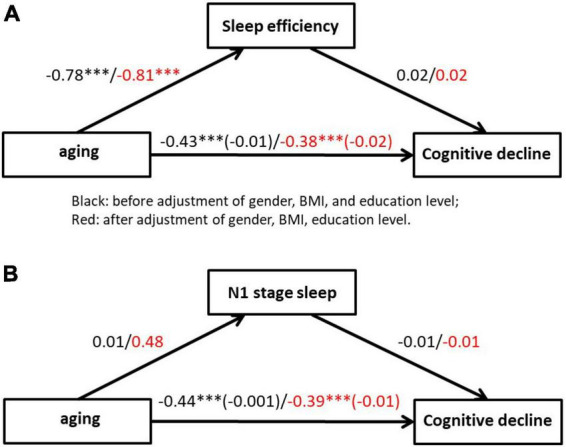
Mediation of the relationship between aging and cognitive decline by mediation effect of sleep efficiency **(A)** and N1 stage sleep **(B)**. Coefficients are standardized regression coefficients that are controlled for gender, BMI and education level. The coefficient in brackets represents the standardized regression coefficient when the mediator is also in the model. ^***^p < 0.001.

Figure 7 represents the regression models testing the relations between aging (independent variable) and sleep efficiency, N1 stage sleep (mediator), and cognitive decline (dependent variable). The regression models revealed that aging was related to higher levels of cognitive decline before (β = −0.43, *P* < 0.001) and after (β = −0.38, *P* < 0.001) adjustment of gender, body mass index (BMI), and education level. Moreover, regression models revealed that aging were also related to sleep efficiency (β = −0.81, *P* < 0.001) control for gender, BMI, and education level.

## 4. Discussion

Age affects both sleep and cognition. The present study showed significant differences in SE and the proportion of N1 stage sleep among people of different ages. Regardless of dementia, SE was higher in the younger group and lower in the older age group. The proportion of N1 stage sleep was lower in the younger group and higher in the older group, regardless of dementia. This indicates that sleep structure changes significantly with age. The minimum OS index was significantly lower in the dementia group than in the cognitively normal group, at both higher and lower ages. In fact, as age increased, SE decreased, whereas the proportion of N1 stage sleep increased. Other studies in healthy individuals have suggested that objectively measured total sleep time, sleep latency, slow wave sleep, wake after sleep onset, and REM sleep decrease through adulthood; however, SE continues to significantly decrease in adults aged 60 years and older (Ohayon et al., 2004).

Among patients with dementia, sleep disorders are associated with poor cognitive function, even in those with mild dementia (Moran et al., 2005). Moreover, disturbed sleep parameters have been linked to an increased risk of the subsequent development of dementia. The deterioration of circadian rhythms begins early in dementia and progresses throughout the disease course (Hatfield et al., 2004). In this study, N1 and SE changed with age and cognitive level of the participants. This suggests that there may be some potential sleep parameters in the younger SD-nCD group that reflect cognitive function. Further analysis of the data from the younger group revealed that patients with greater than average SE had a significantly higher total sleep duration and a significantly lower SWS stage sleep percentage. Patients with N1 stage sleep percentages lower than the mean had a significantly higher N2 stage sleep percentage. According to previous studies, SE is equal to the total sleep time divided by bedtime (Didikoglu and Maharani, 2020). In the SE < SE average condition, N3 sleep duration was significantly prolonged. N3 sleep may be a better indicator of clinical sleep quality than SE sleep. A shorter total sleep time may be related to a longer N3 sleep. There was no feeling of fatigue after sleeping.

AHI and OS were not affected by age but differed significantly in the different cognitive level groups. Sleep parameters can be used as indicators to determine cognitive levels. Regarding the correlation between cognition and sleep, we found that cognitive assessment scores were significantly correlated with the percentage of sleep during SWS and with the minimum OS of sleep. Improvements in sleep quality can also be used to prevent cognitive decline, especially interventions for OSA (Malhotra and White, 2002; Calik, 2016). Targeting modifiable risk factors is critical to reduce the onset and progression of dementia. Depression-related cognitive decline, treated with antidepressant methods, can alleviate sleep disturbances (Gebara et al., 2018). Sleep-disordered breathing is associated with a higher incidence of all-cause dementia, AD, and vascular dementia (Shi et al., 2018).

There is a growing body of literature on sleep and cognitive function in older adults. SD-CD represents neurodegenerative-associated cognitive changes, whereas SD-nCD represents normal- or age-related cognitive changes. Age-related cognitive changes result from developmental maturation. Both SWS and REM sleep decreased with age. In future, the clinical characteristics of patients in the SD-nCD group with sleep structures similar to those in the SD-CD group will be investigated after expanding the sample size. These characteristics could be used as indicators for the early diagnosis of diseases. Moreover, the cognition domain, including memory, attention, and executive functioning, can be considered to explain changes in sleep architecture, fragmentation, quality, and neurological conditions.

Plasma Aβ levels have emerged as a possible predictor of cognitive decline and dementia. This may be modified by health-related factors associated with the risk of dementia. These include insulin resistance and diabetes (Li et al., 2015; Peters et al., 2017), acute cerebral accidents (Kalaria et al., 2016) and impaired sleep (Sanchez-Espinosa et al., 2014; Spira et al., 2014). Epidemiological studies on sleep and cognition have suggested that sleep disorders are a risk factor for cognitive decline and may also be a concomitant disorder of cognitive impairment (Wennberg et al., 2017). Therefore, identifying patients with cognitive decline due to sleep disorders may greatly improve prevention strategies and treatment decisions for cognitive impairment.

Because of the insidious onset of dementia, some early-stage patients do not show cognitive decline but rather sleep disturbances or depressed mood. As the disease progresses, mild cognitive impairment (MCI) gradually appears (Jack et al., 2018). MCI is characterized by cognitive decline with some probability of developing dementia; however, it may also be maintained in MCI. Two-thirds of individuals with either dementia or MCI have sleep disorders (A ncoli-Israel et al., 1991). In addition, there is a physiological decline in sleep quality with age, especially in the length and quality of non-rapid eye-movement sleep. A previous study showed that the risk of AD was 1.68 times higher in patients with sleep disorders than in healthy controls, and more than 60% of patients with MCI and AD had at least one type of sleep disorder (Guarnieri et al., 2012). Previous studies also have shown that elderly patients with sleep disorders not accompanied with dementia have poor executive function due to their poor sleep quality including low sleep efficiency, REM sleep behavior disorder, et al. (Lerche et al., 2018; Boeve et al., 2022). In the future study, exploring the association between sleep disorders and executive functioning is worth investigating. Moreover, although in this study we did not obtain the exact disease course of patients in SD-CD group, we found that previous studies showed sleep abnormalities prior to dementia, usually in the period of mild cognitive decline, which early than dementia many years. The impaired sleep represents one of the earliest symptoms of dementia (Casagrande and Forte, 2022).

Aging can alter both sleep timing and quality, which can be disruptive in AD. Increased production of Aβ and reduced Aβ clearance are caused by the close interplay of Aβ, sleep disturbance, and increased wakefulness. In addition to Aβ, the impact of tau pathology is possibly noteworthy for the sleep deprivation observed in AD. Core AD cerebrospinal fluid biomarkers, including Aβ_42_, total tau, and ptau, can reflect the key elements of AD pathophysiology before the emergence of symptoms (Cui et al., 2022). In future, blood abnormalities in patients with sleep disorders should be investigated in depth to assist in the early diagnosis of dementia. Although the relationship between cause and effect is still ambiguous, there is a strong correlation between aging, sleep disturbance, and cognitive decline, and there may be some indicators in the sleep structure parameters that can help us identify patients with a tendency for cognitive decline at a younger age. Although blood markers have been studied more in cognitive disorders, they have been less in studies of sleep disorders with cognitive decline, and we tried to find some significant differences in blood of three groups in order to find biomarkers suitable for mass screening using sleep indicators combined with blood biomarkers. This minimally invasive approach may significantly improve the sensitivity of early diagnosis which can be used as an effective tool for mass screening.

The limitations of this study are as follows: (1) Comprehensive neuropsychological evaluations were not conducted due to the complexity of clinical process. In this study we adopted MMSE to conduct cognitive assessment. Although MMSE was widely used in screening cognitive impairment, it has low performance in detecting minor neurocognitive disorder or dementia in early stages due to its limited capacity to detect complex cognitive domain disorders. In addition, MMSE has some false-negative results in people with high educational levels. (2) It is unclear whether sleep-related medications have direct effect on cognition. Short-acting sleep medications usually do not lead to next-day drowsiness and have no effect on cognitive assessment; long-acting sleep medications have the potential to cause patients to experience poor daytime functioning in the following day, and may have some effect on cognitive assessment. In this study, many patients used drugs to improve sleep, while the effects of sleep drugs on PSG or cognition were not investigated. We try to recruited patients with consistent medications to improve the confounding factor control. This also led to a relatively small sample size. (3) Although there was association between sleep and cognition disease, the study cannot explain the causality due to it was a retrospective study.

## Data availability statement

The original contributions presented in this study are included in the article/supplementary material, further inquiries can be directed to the corresponding authors.

## Ethics statement

The studies involving humans were approved by the Ethics Committee of Ningbo Kangning Hospital. The studies were conducted in accordance with the local legislation and institutional requirements. Written informed consent for participation in this study was provided by the participants’ legal guardians/next of kin.

## Author contributions

XM and ZZ contributed to the original draft of the manuscript. JW and ZQ conducted the experiments. HY and CZ proofread the manuscript. All authors have read and approved the final manuscript.
